# Visualization of the HIV-1 Env glycan shield across scales

**DOI:** 10.1073/pnas.2000260117

**Published:** 2020-10-22

**Authors:** Zachary T. Berndsen, Srirupa Chakraborty, Xiaoning Wang, Christopher A. Cottrell, Jonathan L. Torres, Jolene K. Diedrich, Cesar A. López, John R. Yates, Marit J. van Gils, James C. Paulson, Sandrasegaram Gnanakaran, Andrew B. Ward

**Affiliations:** ^a^Department of Integrative Structural and Computational Biology, The Scripps Research Institute, La Jolla, CA 92037;; ^b^The International AIDS Vaccine Initiative Neutralizing Antibody Center, The Scripps Research Institute, La Jolla, CA 92037;; ^c^Scripps Consortium For HIV/AIDS Vaccine Development, The Scripps Research Institute, La Jolla, CA 92037;; ^d^Theoretical Biology and Biophysics Group, Los Alamos National Laboratory, Los Alamos, NM 87545;; ^e^Center for Nonlinear Studies, Los Alamos National Laboratory, Los Alamos, NM 87545;; ^f^Department of Molecular Medicine, The Scripps Research Institute, La Jolla, CA 92037;; ^g^Department of Medical Microbiology, Amsterdam University Medical Center, University of Amsterdam, 1105 AZ Amsterdam, The Netherlands;; ^h^Department of Immunology and Microbiology, The Scripps Research Institute, La Jolla, CA 92037

**Keywords:** HIV-1, glycoprotein, cryo-EM, molecular modeling, vaccine

## Abstract

The dense array of N-linked glycans on the HIV-1 envelope glycoprotein (Env), known as the “glycan shield,” is a key determinant of immunogenicity, yet intrinsic heterogeneity confounds typical structure–function analysis. Here, we present an integrated approach of single-particle electron cryomicroscopy (cryo-EM), computational modeling, and site-specific mass spectrometry (MS) to probe glycan shield structure and behavior at multiple levels. We found that dynamics lead to an extensive network of interglycan interactions that drive the formation of higher-order structure within the glycan shield. This structure defines diffuse boundaries between buried and exposed protein surface and creates a mapping of potentially immunogenic sites on Env. Analysis of Env expressed in different cell lines revealed how cryo-EM can detect subtle changes in glycan occupancy, composition, and dynamics that impact glycan shield structure and epitope accessibility. Importantly, this identified unforeseen changes in the glycan shield of Env obtained from expression in the same cell line used for vaccine production. Finally, by capturing the enzymatic deglycosylation of Env in a time-resolved manner, we found that highly connected glycan clusters are resistant to digestion and help stabilize the prefusion trimer, suggesting the glycan shield may function beyond immune evasion.

## Significance

The HIV-1 Env “glycan shield” masks the surface of the protein from immune recognition, yet intrinsic heterogeneity defies a typical structure–function description. Using an integrated approach of cryo-EM, computational modeling, and mass spectrometry, we visualized the glycan shield structure in a new light. Our approach facilitated development of cryo-EM analysis methods and allowed for validation of models against experiment. Comparison of Env expressed in different cell lines revealed how subtle differences in composition impact glycan shield structure and affect the accessibility of epitopes on the surface, providing insights for vaccine design. Finally, time-resolved cryo-EM experiments uncovered how highly connected glycan clusters help stabilize the prefusion trimer, suggesting the glycan shield may function beyond immune evasion.

The HIV type 1 (HIV-1) envelope glycoprotein (Env) is the sole antigen on the surface of the virion and has evolved several tactics for evading the adaptive immune system, chief among which is extensive surface glycosylation (1–3). Env has one of the highest densities of N-linked glycosylation sites known, with glycans accounting for ∼1/2 the mass of the molecule (4–8). This sugar coat, or “glycan shield,” is common among viral fusion proteins and is believed to be a primary hurdle in the development of neutralizing antibodies against Env during infection and vaccination (9, 10). Therefore, arriving at a consistent and general description of its structure and dynamics may prove necessary in designing an effective Env-based immunogen and be of broader importance toward understanding the structure and function of densely glycosylated proteins in general.

N-linked glycans can be highly dynamic, however, and can vary substantially in length, connectivity, and chemical composition (11, 12). On the Env ectodomain surface there are ∼95 glycosylation sites on average (6), and mass spectrometry (MS) analysis has revealed that compositional heterogeneity exists between sites as well as at the same site between different copies of Env (13). While heterogeneity and dynamics are critical for immune evasion, they are incompatible with a typical structure–function description. When the first structures of the fully glycosylated Env were solved with single-particle electron cryomicroscopy (cryo-EM) (14, 15), most glycans were not resolved beyond the first or second sugar ring except when stabilized by antibodies. This suggested that the glycan shield is highly dynamic; however, due to the missing high-resolution details, structural analysis remained focused on Env–antibody interactions, while experimental investigations into the glycan shield shifted primarily toward chemical analysis via MS (11). One crystallography study specifically addressing glycan shield structure was recently published and the data show evidence for highly stabilized glycans engaging in a wide range of interglycan contacts (6), which is at odds with the cryo-EM results. Such a disagreement between the two techniques highlights the difficulty in studying the glycan shield and the need for more focused experiments.

Computational modeling, in particular molecular dynamics (MD) simulations, has proven useful as a complementary approach for characterizing glycoprotein structure and dynamics (16–19), including Env (6, 20–22). Three sets of atomistic dynamics simulations of fully glycosylated Env trimers were recently reported and these predicted that dynamics would lead to extensive shielding of the antigenic surface and the formation of extensive networks among interacting glycans (6, 21, 22). In one study, glycans were observed clustering into distinct microdomains over long timescales, and it was found that neutralizing antibodies preferentially target the interfaces between these domains, suggesting the glycan shield may possess biologically relevant large-scale structure. Although MD is a widely accepted method for modeling glycoproteins, atomistic simulations for systems as large as Env require substantial computational resources to access biologically relevant spatial and temporal scales and can suffer from initial model bias. Therefore, more computationally efficient methods for modeling fully glycosylated Env are needed. Furthermore, there are still no established methods for experimentally validating the results of these simulations.

Here, we describe an integrated approach combining cryo-EM, computational modeling, and site-specific MS aimed at illuminating glycan shield structure and behavior at multiple levels. First, we introduce a model system for our cryo-EM experiments and describe the complete structure of the native Env glycan shield. In parallel, we developed a high-throughput modeling pipeline for rapidly generating diverse ensembles of fully glycosylated Env at atomistic resolution, enabling quantification of glycan-specific geometric properties and concerted behavior within the glycan shield as a whole. We then used these ensembles as ground truth for the creation of simulated cryo-EM data, which facilitated the development of analysis tools and enabled the validation of theoretical models against cryo-EM experiments.

With our integrated approach, we show that the glycan shield is highly dynamic but exhibits variation in dynamics between glycans due in part to crowding and other geometric and energetic constraints. We found that dynamics give rise to a network of interglycan interactions that drive the formation of higher-order structure within the glycan shield. This blurry low-resolution structure creates diffuse boundaries between buried and exposed protein surface that define potential sites of vulnerability. Using Env expressed in three common cell lines, we show how differences in glycan composition and occupancy can be detected by cryo-EM and result in changes to glycan shield structure and dynamics that affect the accessibility of epitopes on the surface. To complement our results, we present site-specific mass spectrometry data for the same samples. Finally, by exposing Env to endoglycosidase digestion and capturing reaction intermediates with cryo-EM in a time-resolved manner, we found that highly connected glycan clusters are resistant to digestion and act to stabilize the prefusion trimer structure, implying the glycan shield may function beyond immune evasion.

## Results

### Env in Complex with a Base-Specific Antibody as a Model System for Cryo-EM Analysis of the Native Glycan Shield.

**Fig. 1.**
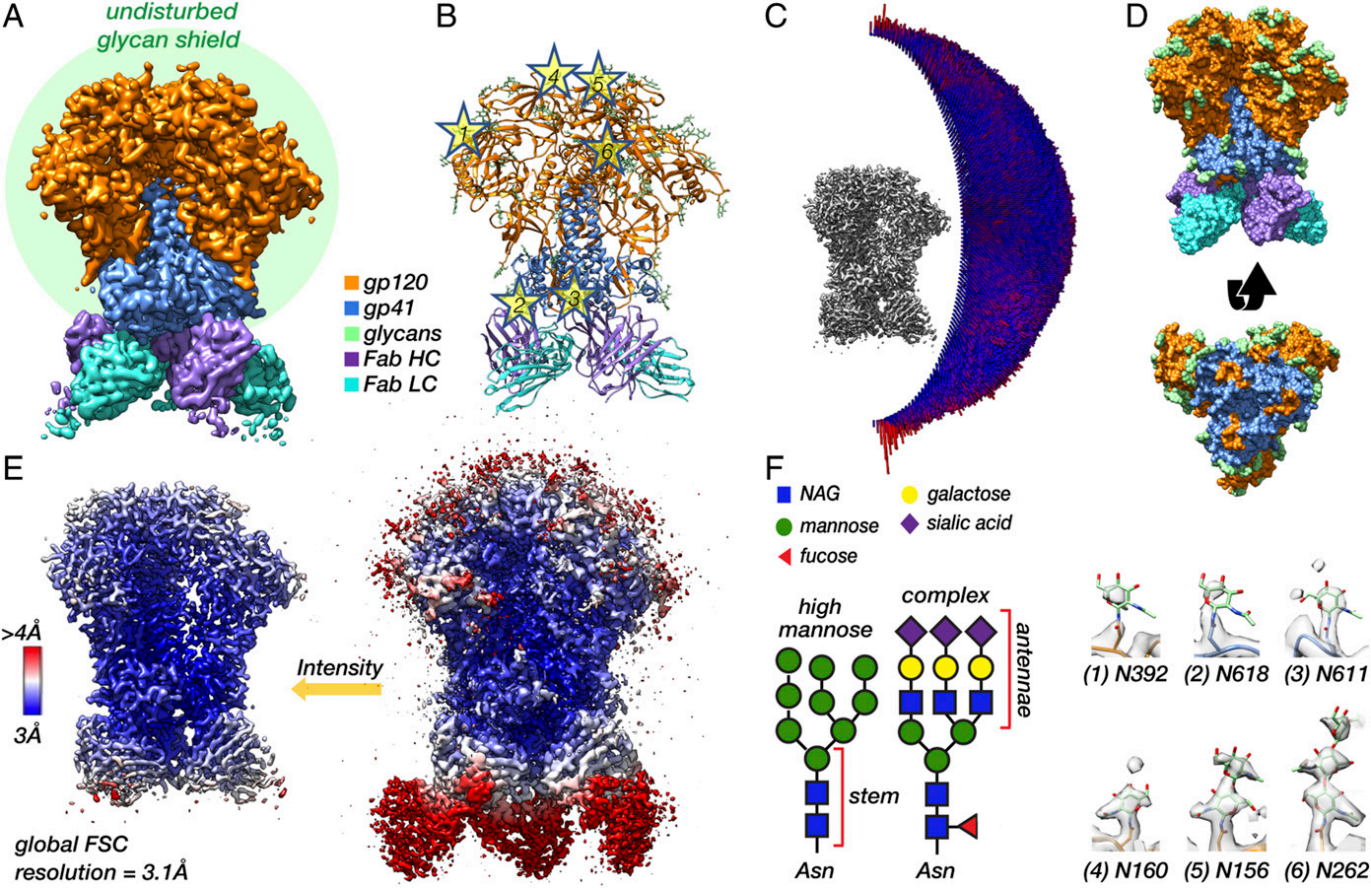
Soluble SOSIP in complex with a base-specific Fab as a model system for cryo-EM analysis of the native HIV-1 Env glycan shield. (*A*) Segmented cryo-EM map of BG505 SOSIP.664 in complex with three copies of the RM20A3 Fab. The green circle is meant to represent the glycan shield. HC, heavy chain; LC, light chain. (*B*) Refined atomic model with stars highlighting several N-linked glycans. (*C*) Three-dimensional angular distribution histogram. (*D*) Surface representation of the refined atomic model viewed from the side and from the bottom with the Fab chains removed. (*E*) Sharpened 3.1-Å-resolution cryo-EM map at high and low threshold colored by local resolution. (*F*) Symbol nomenclature for glycan depiction (74) of a representative high-mannose and complex-type glycan (*Left*). Cryo-EM map density for six representative glycans from the refined atomic model (numbers corresponding to the stars in *B*).

We chose BG505 SOSIP.664 as a model system to demonstrate our approach because it is the first and most widely studied of the soluble prefusion stabilized Envs (23, 24). BG505 SOSIP.664 is also currently in the first ever human clinical trials of a trimeric subunit-based HIV vaccine (https://clinicaltrials.gov/ct2/show/NCT03699241). In Fig. 1, we present the cryo-EM reconstruction and refined atomic model of BG505 SOSIP.664 purified from human embryonic kidney 293F (HEK293F) cells in complex with three copies of the fragment antigen-binding domain (Fab) of the nonneutralizing antibody RM20A3. This complex will be referred to as BG505_293F from here on. The RM20A3 Fab was included because it improves orientation bias (Fig. 1*C*), and binds to a nonnative epitope at the base of the SOSIP trimer, thus leaving the native glycan shield undisturbed. (Fig. 1*D*).

The global resolution of this C3-symmetric reconstruction determined by Fourier shell correlation (FSC) is ∼3.1 Å (*SI Appendix*, Fig. S1*A*), and the bulk of the protein is at or near this resolution (Fig. 1*E*). Approximately 80% of Env residues could be confidently built into this map, while most glycans are ill-defined beyond the core N-acetylglucosamine (NAG), accounting for only ∼15% of the total predicted glycan mass (Fig. 1). Glycans located on the poorly resolved flexible loops (N185e, N185h, N398, N406, N411, and N462) could not be identified at all; however, site-specific MS data confirmed they are indeed glycosylated (25) (*SI Appendix*, Fig. S2*A*). N-linked glycans on Env can be roughly classified into two types, high-mannose and complex, based on the extent of intracellular processing, and they differ in composition and structure (*SI Appendix*, Fig. S1*F*). Even if the glycans were better resolved, averaging of individual projections during three-dimensional (3-D) reconstruction means we cannot uniquely identify the specific type of glycan at most sites because they possess a mixture of several different types (26) (*SI Appendix*, Fig. S2*A*). Of those we could identify, a few were found to be near-uniformly processed (>75% complex), for example the glycans at N611 and N618 on gp41, and it should be possible to confirm this by the presence of a core fucose residue. However, we were unable to identify clear density for fucose at any of these sites (Fig. 1*F*). Together, these observations show that the glycan shield is highly dynamic with respect to the protein core but exhibits some differences in ordering between glycans, and that it is not possible to distinguish between the different types of glycans from a single cryo-EM map when the glycans are not stabilized by antibodies.

### Scale-Space and 3-D Variability Analysis Reveals Interconnectivity and Higher-Order Structure within the Glycan Shield.

**Fig. 2.**
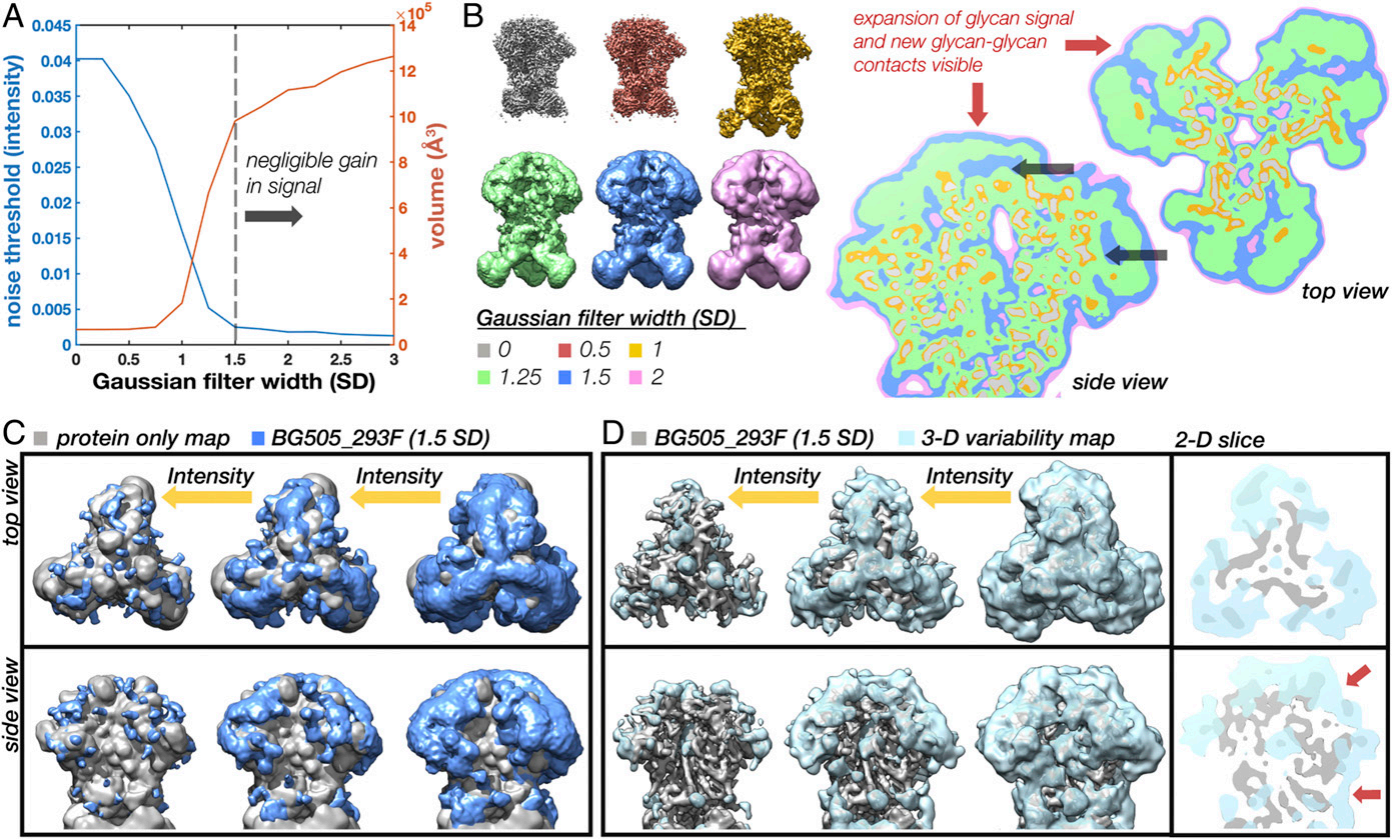
Scale-space and 3-D variability analysis reveals a low-resolution structure in the glycan shield. (*A*) Noise threshold and volume at the noise threshold as a function of Gaussian filter width measured in SDs showing the emergence of glycan signal at low resolutions and plateau around 1.5 SD. (*B*) BG505_293F cryo-EM map at six representative scales along with 2-D slices through the maps overlaid on top of one another showing the emergence of glycan signal and new glycan–glycan contacts visible at low resolutions and thresholds (red arrows). Black arrows are pointing to protein surfaces occluded by glycans. (*C*) Gaussian filtered (1.5 SD) BG505_293F map (blue) visualized at three intensity thresholds along with a protein-only map (gray). (*D*) SPARX 3-D variability map (light blue) visualized at three intensity thresholds along with the BG505_293F map (gray) and 2-D slices through the top and side of the map with red arrows highlighting glycan–glycan contacts.

Although we lack the resolution to build precise atomic models, there is still a great deal of information about glycan shield structure and dynamics that can be gleaned from the cryo-EM data. At low isosurface thresholds, noise appears surrounding Env’s well-resolved protein core where the missing glycan mass should be (Fig. 1*E*). To look for meaningful structure within this noisy signal, we analyzed properties of the map across a range of scales by progressively smoothing it with a Gaussian filter of increasing standard deviations (SD). Resolution in the glycan shield falls off with distance from the protein surface; therefore, as the map is smoothed, more and more glycan signal should become visible until the map encompasses the majority of the space occupied by the ensemble of configurations. This process could be captured by measuring the volume of the map across the scale space. To calculate the volume, however, one must first choose an appropriate threshold. For our analysis, we implemented an autothresholding method based on a simple measure of topological connectivity described in *SI Appendix*, Fig. S3 *A*–*F*. We refer to the resulting intensity as the “noise threshold” since it represents the lowest threshold before the appearance of noise. Fig. 2*A* shows the noise threshold and volume of the map at the noise threshold as a function of Gaussian filter width. Both curves have a sigmoidal shape; once the map is sufficiently smoothed it undergoes a period of rapid expansion in volume and reduction in noise threshold that is presumably capturing the appearance of signal from the poorly resolved regions of the map. The curves then plateau around ∼1.5 to 2 SD, suggesting there is negligible gain in signal with further smoothing.

Examination of these filtered maps confirms the progressive emergence of more structure in the glycan shield (Fig. 2*B*). By taking 2-D slices through six representative maps and displaying them on top of one another, we see how glycosylated surfaces undergo substantially more dilation (red arrows) than the nonglycosylated surfaces. Once the isosurface has expanded to include signal from glycan antennae, neighboring glycans begin to merge, creating a canopy-like effect that occludes underlying protein surfaces (black arrows). This implies that neighboring glycans sample overlapping volumes and have the potential to interact. Also apparent is the negligible gain in signal moving from 1.5 to 2 SDs (blue and pink). We therefore concluded that the 1.5-SD Gaussian filtered map is the ideal scale for interpreting glycan shield structure in this reconstruction. Visualization of this filtered map across a range of thresholds reveals a hierarchy of structural features (Fig. 2*C*). At high thresholds, the glycan shield is composed primarily of isolated and well-defined glycan stems, while the majority of the protein surface is exposed (gray map). Moving to lower thresholds, it progresses through a series of higher-order structural states defined by isolated clusters of multiple overlapping glycans, until finally becoming completely interconnected near the noise threshold. This low-threshold structure occludes large regions of protein surface and represents the full extent of the glycan shield.

As a complementary approach for visualizing the dynamic glycan shield, we performed 3-D variability analysis in the SPARX software package (27, 28) (Fig. 2*D*). Three-dimensional variability, which is closely related to 3-D variance, should be high anywhere there is significant heterogeneity in the map. Indeed, we see high variability around known flexible regions such as the constant domains of the Fabs (clipped from view) as well as variable loops and the exterior of the protein surface at the sites of N-linked glycans. Variability in the glycans is highest at the distal ends of the glycan stems and expands outward as the threshold is reduced, revealing extensive interconnectivity (Fig. 2*D*). Two-dimensional slices through the variability map highlight the canopy effect between neighboring glycans, which acts to occlude underlying protein surfaces (red arrows in Fig. 2*D*). Overall, the shape and topology are similar to the Gaussian filtered map. A more complete description of the 3-D variability can be found in *SI Appendix*.

### A High-Throughput Atomistic Modeling Pipeline for Generating Large Ensembles of Glycosylated Env.

**Fig. 3.**
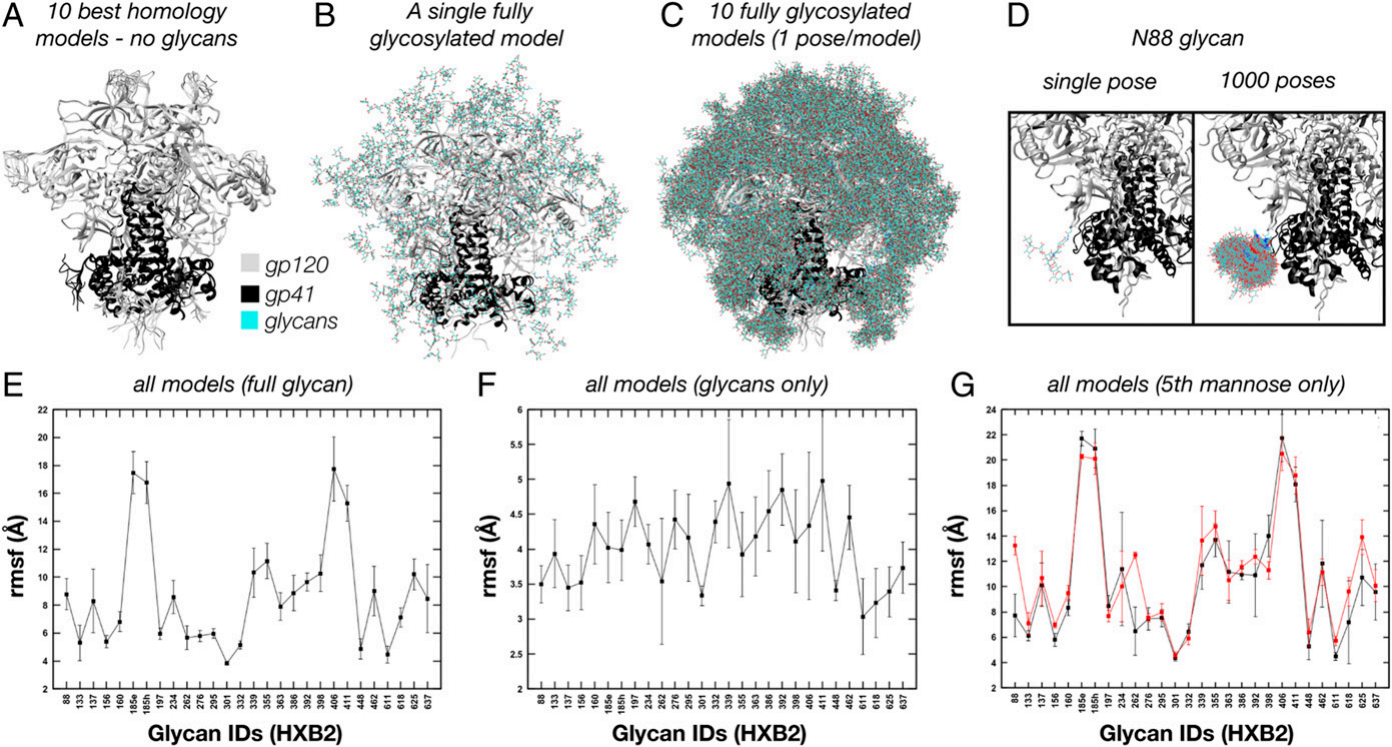
ALLOSMOD-based HT-AM pipeline for fast and robust sampling of fully glycosylated Env. (*A*) Ten homology models used as protein scaffolds. (*B*) One of the 10 models with a single relaxed Man9 glycan at each site. (*C*) One fully glycosylated and relaxed model for each of the 10 protein scaffolds. (*D*) Close-up of the N88 glycan showing a single glycan pose (*Left*) and all 1,000 poses (*Right*). (*E*) Full glycan average rmsf for all 1,000 poses. (*F*) Glycan-only rmsf for all 1,000 poses. (*G*) Full glycan average rmsf for all 1,000 poses at the fifth mannose residue for both the Man9 and Man5 ensembles.

Cryo-EM does not capture atomic details of individual molecules, so to better understand the results presented above, we performed atomistic simulations of fully glycosylated BG505 SOSIP.664. To overcome the sampling limitations of MD simulations, we employed a high-throughput atomistic modeling (HT-AM) pipeline based around the ALLOSMOD package of the MODELLER software suite (29), which has been used previously to generate small ensembles of glycosylated Env (30). This methodology accounts for both energetic and spatial constraints on glycan sampling by a combination of empirical energy minimization-based structural relaxation and simulated annealing [described in detail by Guttman et al. (29)], along with initial randomization of glycan orientations. However, it does not provide temporal information about dynamics. Here, we have built on this by incorporating 10 different protein scaffolds and additional variability in the V2 and V4 loops (Fig. 3*A*) and utilized it to generate much larger ensembles of 1,000 fully glycosylated Env structures with uniform mannose-9 (Man9) glycans. Ten such models are shown in Fig. 3*C*, one from each protein scaffold, while the full set of models at a single glycosylation site is shown in Fig. 3*D*. We also repeated the simulation with uniform mannose-5 (Man5) glycosylation for comparison.

### The HT-AM Pipeline Captures Spatial and Energetic Constraints on Glycan Flexibility.

To assess how spatial and energetic constraints affect the flexibility of individual glycans in our simulations, we calculated the root-mean-squared fluctuation (rmsf) for each glycan across all 1,000 models after aligning the protein scaffold. Our method captures variability between glycans (Fig. 3*E*), and the additional flexibility imparted by the 10 starting protein scaffolds can be appreciated by comparison with the glycan-only rmsf (Fig. 3*F*). For example, glycans located on the flexible loops have a much higher rmsf than all other glycans (185e, 185h, 406, and 411) whereas glycans at the N262, N301, N332, N448, and N611 sites have lower rmsf. This leads to a large difference in sampled volumes between the most and least dynamic glycans (*SI Appendix*, Fig. S4*A*). We also see an increase in average rmsf of the individual glycan residues starting from the core NAG and moving outward to the tips of each antenna (*SI Appendix*, Fig. S5 *A*–*C*), which is in line with the cryo-EM results showing reduced resolution beyond the core NAG (Fig. 1*D*).

We observed a similar trend for the Man5 ensemble (*SI Appendix*, Fig. S5 *G* and *H*); however, by comparing the average rmsf values of the fifth mannose residue alone, we found a slight increase in rmsf and sampled volume at most sites compared with the Man9 ensemble (Fig. 3*G* and *SI Appendix*, Fig. S5 *I* and *J*). We attribute this effect to increased crowding in the glycan canopy from the more massive Man9 glycans. In support of this, there is a significant positive correlation between glycan flexibility and glycan crowding (*SI Appendix*), however only when considering relatively large neighborhoods (*SI Appendix*, Fig. S6*A*). A similar trend was observed by Stewart-Jones et al. which they attributed to different “shells” of influencing glycans (6). Such higher-order dynamic effects could be possible in light of our structural observations.

In addition to crowding effects, the local protein structure will also influence glycan dynamics. In our modeling pipeline, the protein backbone was kept harmonically restrained close to the template to allow for extensive sampling of glycan conformations using simulated annealing. Thus, we see that the Asn side chains of residues 88, 160, 197, 234, and 262 all have very low rmsf (*SI Appendix*, Fig. S5*F*), possibly stemming from the limited torsional space available during modeling. The glycosylated Asn residues in gp41 have relatively low rmsfs as well (N611, N618, N625, and N637), being situated on stable helical bundles (*SI Appendix*, Fig. S4*B*). This ultimately results in a relative reduction of the glycan dynamics at some of these sites (Fig. 3*C*). Correcting for the contribution to fluctuations coming from the underlying protein, we observed that the rmsfs between the different glycans are comparable, ranging from 3 to 5 Å, with a similar scale of SD (Fig. 3*F*).

### Simulated Cryo-EM Maps Reproduce Defining Features of the Experimental Data.

**Fig. 4.**
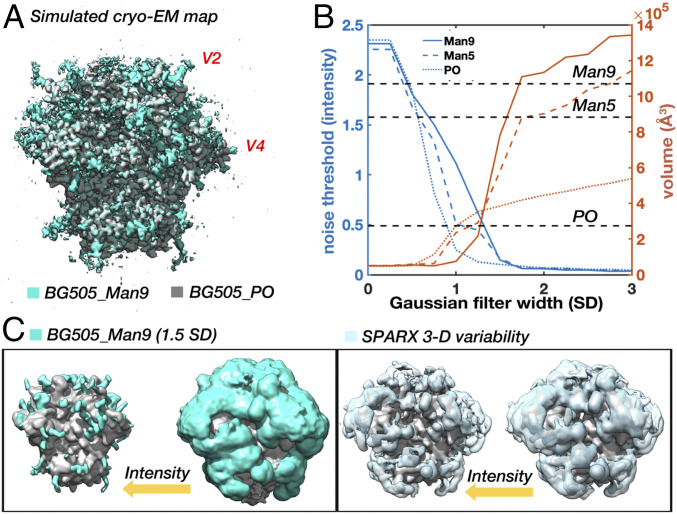
Simulated cryo-EM maps from the HT-AM ensembles reproduce defining features of the experimental data. (*A*) Simulated cryo-EM map generated from the Man9 ALLOSMOD ensemble (teal) and a protein-only (PO, gray) ensemble. Each of the 1,000 models was projected at 100 uniformly distributed angles with added white noise and reconstructed in Relion with C3 symmetry then sharpened with a data derived B-factor. (*B*) Noise threshold and volume at the noise threshold as a function of Gaussian filter width (SD) for the BG505_Man9, BG505_Man5, and BG505_PO simulated cryo-EM maps with dashed lines indicating the volume sampled by each ensemble. (*C*) Gaussian filtered (1.5 SD) BG505_Man9 (teal) and BG505_PO (gray) maps along with the SPARX 3-D variability map (light blue) viewed at high- and low-intensity thresholds.

Given the single-molecule nature of cryo-EM datasets, the ensembles generated by the HT-AM pipeline can be seen as representing the individual particles that go into a 3-D reconstruction. With that in mind, we established a protocol for transforming the ensembles into simulated cryo-EM datasets (*SI Appendix*, Fig. S7*A*). For comparison, we replicated the process for the Man5 ensemble as well as a protein-only (PO) ensemble (BG505_Man9, BG505_Man5, and BG505_PO, respectively). The simulated maps reproduced some of the defining features of the experimental data. For instance, refinement was dominated by the stable protein core and only the first few sugar residues at each site are defined when the map is filtered to the global FSC resolution (Fig. 4*A*). This leads to a similar scale-space structure (Fig. 4*B*), with a plateau again appearing around 1.5 to 2 SDs when the maps are filtered to the same initial resolution, suggesting that the HT-AM pipeline is capturing physiologically relevant sampling in the glycan shield, at least globally. Importantly, we found that the volume of the 1.5-SD Gaussian filtered map at the noise threshold closely approximates the total volume sampled by the ensemble (Fig. 4*B*, dashed lines). Comparing the curves for all three simulated reconstructions (Fig. 4*B*), we see how differences in glycosylation manifest as differences in volume at low resolutions, suggesting cryo-EM can be used to capture global changes in glycan shield composition between two reconstructions of the same Env. Visualization of the 1.5-SD Gaussian filtered BG505_Man9 map at high and low thresholds reveals a similar evolution toward a more connected topology and extensive shielding of the protein surface at low thresholds (Fig. 4*C*). We also calculated the 3-D variability map for the BG505_Man9 reconstruction and observed a similar result to the experimental data (Fig. 4*C*). In addition, we compared the SPARX variability map with the true 3-D variance (*SI Appendix*, Fig. S7*B* and *C*) and found negligible differences between the two (see *SI Appendix* for details).

### Measuring Glycan Dynamics in Cryo-EM Maps.

**Fig. 5.**
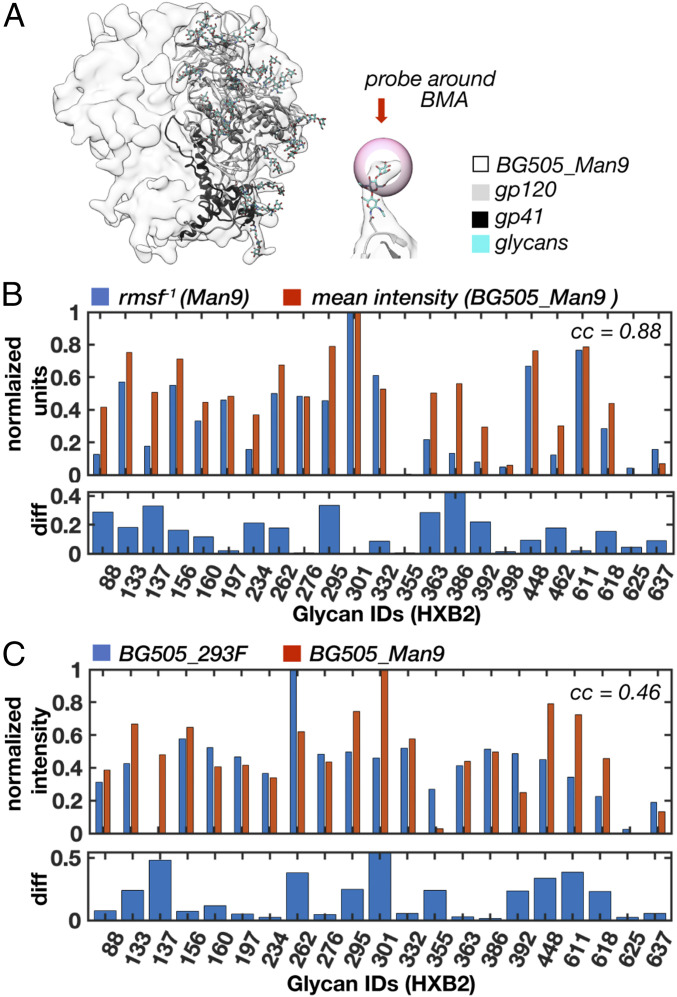
Measuring glycan dynamics from cryo-EM maps reveals close agreement between simulation and experiment. (*A*) Gaussian filtered (1.5 SD) BG505_Man9 simulated cryo-EM map (transparent) and atomic model used for local intensity analysis. Also shown is a single glycan stem with a spherical probe around the BMA residue. (*B*) Normalized mean intensity around BMA residues for BG505_Man9 (probe radius 2.3 Å) and the inverse normalized full glycan average rmsf. Pearson correlation coefficient ∼0.88 (*P* value = 2.1e-8). (*C*) Normalized mean intensity around BMA residues for BG505_293F and BG505_Man9 along with the absolute difference at each site. Pearson correlation coefficient ∼0.46 (*P* = 0.03).

Measuring intensity around individual glycans in the cryo-EM maps should allow us to assess their relative dynamics. To confirm this, we used the simulated BG505_Man9 map since we could make direct comparison with the rmsf values. However, only the first one or two glycan residues are defined at most sites in the high-resolution map (Fig. 4*A*) and their dynamics deviate significantly from the rest of the glycan (*SI Appendix*, Fig. S8*A*). The third glycan residue, β-mannose (BMA), more closely approximates the average rmsf (mean deviation ∼2 Å) and the relative differences between sites (*SI Appendix*, Fig. S8*B*). In addition, the glycan stems are well-defined in the 1.5-SD Gaussian filtered map, which allowed us to identify their locations in the map with reasonable accuracy. So, we built and relaxed glycan stems into the 1.5-SD Gaussian filtered BG505_Man9 simulated map (Fig. 5*A*) at every glycosylation site we could confidently identify (23/28 per protomer). We could not identify the V2 and V4 loop glycans at N185e, N185h, N406, and N411, which had the highest rmsf values in the simulation, nor the glycan at N339, which has a lower rmsf but projects directly toward the heterogeneous V4 loop. The positions of the BMA residues were then used to analyze local map statistics within a spherical probe around each glycan (Fig. 5*A* and *SI Appendix*, Fig. S8*C*). We observe a strong positive correlation between the inverse rmsf and normalized mean intensity (Fig. 5*B*), with a correlation coefficient of ∼0.88 (*P* = 2.1e-8). Thus, local intensity around BMA residues accurately captures ground-truth differences in relative dynamics between glycans and can be used to make direct comparisons between the simulated and experimental cryo-EM maps.

### The HT-AM Pipeline Reproduces Physiologically Relevant Trends in Glycan Dynamics Measured by Cryo-EM.

To make direct comparisons with the experimental data, we built and relaxed glycan stems into the 1.5-SD Gaussian filtered BG505_293F map as described above and could identify clear density at 21/28 glycosylation sites per monomer, two fewer than from the simulated map (N398 and N426). The other five missing glycans were common to both maps, meaning the HT-AM pipeline captured physiologically relevant dynamics at these sites, at least up to the detection limits of this method. Overall, we found that the HT-AM pipeline captured a similar trend in glycan dynamics with a correlation coefficient between the two of ∼0.46 (*P* = 0.03) (Fig. 5*D*).

In addition to the V4 and V5 loop glycans at N398 and N462, we also saw a large deviation at the N137 glycan on the V1 loop, as well as the N262 and N301 glycans. In the BG505_293F map, the N262 glycan was the most ordered due to stabilizing contacts with the gp120 core, and these interactions may not have been accurately captured by the simulation given the restricted protein dynamics. In gp41, a large deviation also occurred at the N611 glycan, which we attribute to restrictive sampling of the protein backbone as previously discussed. To complicate the comparison, the N618 and N625 sites are underoccupied as revealed by MS (*SI Appendix*, Fig. S2*A*). Although suboccupancy will cause reduced signal intensity due to averaging, the severity of this effect is diminished when dynamics at that site are large relative to other glycans, which is the case for the glycan at N625.

### Detecting Site-Specific Changes in Glycan Dynamics, Occupancy, and Chemical Composition from Cryo-EM Maps.

**Fig. 6.**
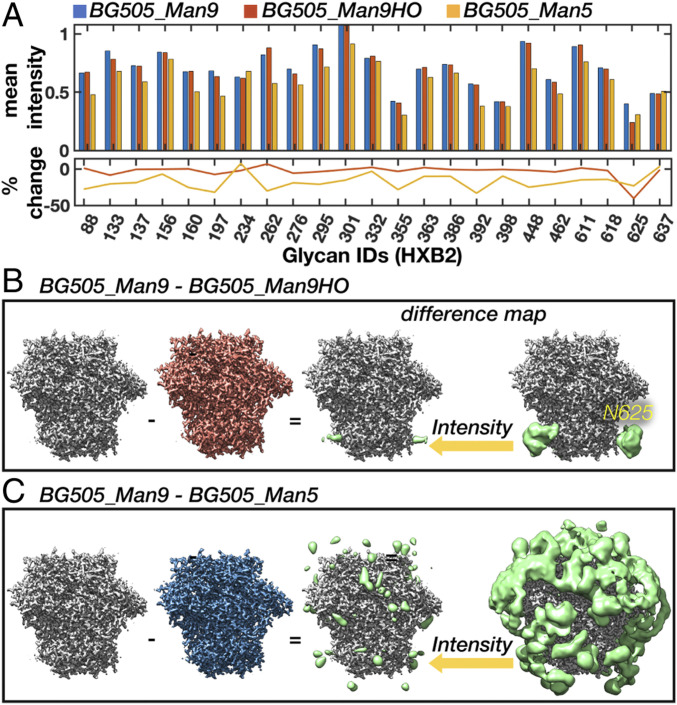
Detecting changes in glycan dynamics, occupancy, and chemical composition from simulated cryo-EM maps. (*A*) Mean intensity around each glycan BMA residue for the BG505_Man9, BG505_Man5, and BG505_Man9HO maps along with the percent change from BG505_Man9. (*B* and *C*) BG505_Man9 − BG505_Man9HO (*B*) and BG505_Man9 − BG505_Man5 (*C*) difference maps (Gaussian filtered) at two intensity thresholds (*Right*; green) along with high-resolution sharpened maps (*Left*).

To test whether these methods could be used to detect site-specific changes in dynamics and occupancy between cryo-EM maps of differentially glycosylated Env, we performed a comparative analysis between the BG505_Man9 and BG505_Man5 simulated maps. Given that the stems of a Man9 and Man5 glycan are identical, the only changes in intensity around the BMA residue should arise from differences in dynamics alone. On average, we see an ∼17% reduction in intensity indicative of increased dynamics, which is in line with the rmsf data (Fig. 6*A*). We also accurately detect the largest increase and decrease in dynamics at the N262 and N234 sites, respectively. To verify we could detect changes in occupancy, we removed the glycan at the N625 site from half of the models and rerefined the data (referred to as BG505_Man9HO for “half-occupancy”). Not surprisingly, we see an ∼50% reduction in mean intensity from the fully occupied reconstruction around this site (Fig. 6*A*).

Another technique that should be sensitive to subtle changes between two cryo-EM maps is difference mapping. Indeed, the change in occupancy at the N625 site is apparent in the BG505_Man9 − BG505_Man9HO difference map (Fig. 6*B*). At high threshold, the signal is localized around the glycan stem and extends to the protein surface. In the BG505_Man9 − BG505_Man5 difference map (Fig. 6*C*), however, the difference signal is strongest where the distal tips of the Man9 glycans would be and does not extend to the protein surface. These results establish cryo-EM as a tool for measuring glycan dynamics as well as changes in chemical composition and occupancy between differentially glycosylated Envs.

### Insights Gained from Analysis of Simulated Data Allow Improved Characterization of Cell Type-Specific Differences in the Glycan Shield.

**Fig. 7.**
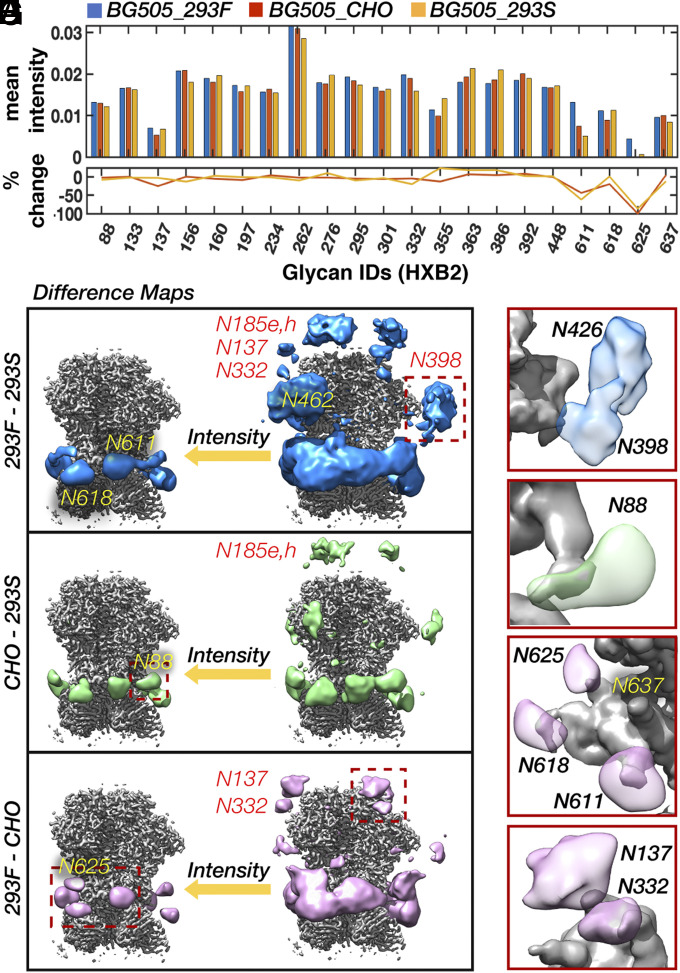
Detection of cell type-specific changes in glycan shield composition and dynamics with cryo-EM. (*A*) Mean intensity at each glycan BMA residue for the BG505_293F, BG505_293S, and BG505_CHO cryo-EM maps along with the percent change from BG505_293F. (*B*–*D*) BG505_293F − BG505_293S (*B*), BG505_CHO − BG505_293S (*C*), and BG505_293F − BG505_CHO (*D*) difference maps at two intensity thresholds. All difference maps were multiplied by a soft mask around the RM20A3 Fabs and then smoothed with a 2-SD Gaussian filter. (*E*–*H*) Flyouts of the regions outlined by dashed red boxes in *B*–*D*.

To test our methods experimentally, we collected cryo-EM data on complexes of RM20A3 Fab and BG505 SOSIP.664 expressed in two additional common cell lines that produce major and minor changes in glycosylation: HEK293S cells and a stable Chinese hamster ovary (CHO) cell line (referred to as BG505_293S and BG505_CHO, respectively). The stable CHO cell line-expressed Env sample was provided to us by the International AIDS Vaccine Initiative (IAVI) as part of the demo run conducted prior to the currently ongoing human clinical trials, and should be identical to the final vaccine product (31). Both datasets refined to ∼3-Å resolution (*SI Appendix*, Fig. S1*A*) and the three atomic structures are nearly identical (Cα rmsds ∼1 Å in *SI Appendix*, Fig. S1*B*). Scale-space (*SI Appendix*, Fig. S3 *G*–*I*) and 3-D variability analysis (*SI Appendix*, Fig. S9 *A*–*C*) also revealed similar low-resolution structural features to BG505_293F; however, comparison of local map intensity around BMA residues uncovered some significant difference in the gp41 glycans, particularly at the N611 and N625 sites, potentially from changes in occupancy (Fig. 7*A*). This is further supported by site-specific MS data collected for the two additional samples (*SI Appendix*, Fig. S2*A*).

The 293S sample contains only high-mannose–type glycans and should therefore allow detection of complex glycans via difference mapping and, to a lesser extent, different distributions of high-mannose glycans. Indeed, we see strong difference signal around the primarily complex gp41 glycans in both the difference maps (Fig. 7 *B* and *C*). We also observe signal around the primarily complex glycan at N88 (Fig. 7*F*). In addition, clear difference signal appeared around the V2, V4, and V5 loops, specifically near the glycans at N185e and N185h, N398, and N462, all of which are shown to be complex by MS. The MS data also show a subtle difference in both occupancy and percentage of complex glycans at the N398 site between the 293F and CHO samples, which could explain why there is strong difference signal in one map and not the other. According to the MS data, the only significant differences in occupancy between the 293F and 293S samples occurred at the N137, N133, and N611 sites; however, the difference maps do not contain any signal around the N611 glycan, suggesting there may be discrepancies between the two methods.

The MS analysis also detected differences in occupancy between the CHO sample compared with the 293F sample at multiple sites (*SI Appendix*, Fig. S2*A*). Indeed, upon closer examination, we found that the difference signal around the gp41 glycans at N611, N618, and N625 extends all of the way to the protein surface (Fig. 7*G*), indicative of changes in occupancy. In this difference map, there is also clear signal around the N137 site, and to a lesser extent at the tip of the N332 glycan stem (Fig. 7*H*). Given the proximity of N137 and N332, it is plausible that suboccupancy at one is driving changes in dynamics and/or glycan distribution at the other. Similar higher-order effects have also been observed via MS when comparing glycoform distributions on Env before and after knocking out specific glycans (26, 32, 33).

### A Probabilistic Glycan–Glycan Interaction Network Reveals Highly Connected Glycan Clusters.

**Fig. 8.**
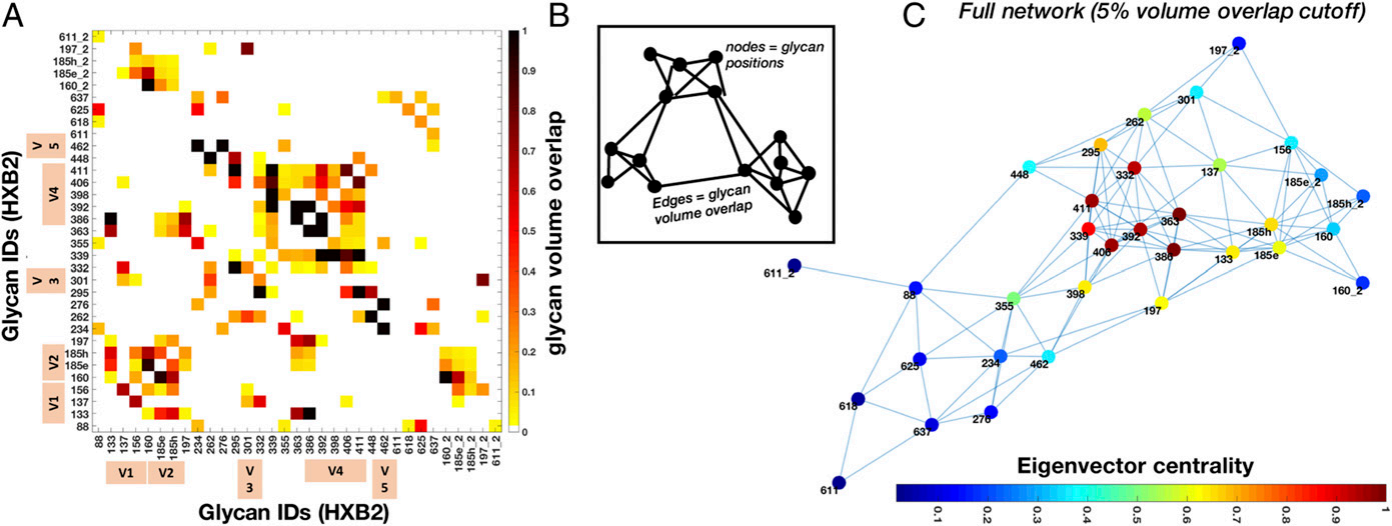
Probabilistic glycan–glycan interaction network for the Man9 ensemble. (*A*) Glycan–glycan volume overlap matrix. Interprotomer overlap is given by the suffix “_2” and the variable loop glycans are indicated by tan color bars. The overlap fraction is normalized with at least 50% overlap being designated as 1. (*B*) Cartoon model of the glycan–glycan interaction network generated by interpreting the overlap matrix as an adjacency matrix. The edge length is drawn inversely proportional to the overlap value. Edges represent the overlap between two glycans represented by nodes. (*C*) Glycan–glycan interaction network calculated from the matrix in *A* with nodes colored by normalized eigenvector centrality.

Both the experimental and simulated cryo-EM maps showed extensive interconnectivity among glycans, so we sought to quantify this more precisely. With each glycan in our models sampling a particular region of space, neighboring glycans can explore overlapping volumes, and the fraction of this overlap gives a measure of their interaction probability. Fig. 8*A* shows a heatmap of the normalized glycan–glycan volume overlap matrix. By interpreting this as an adjacency matrix, we can model the glycan shield as a network. Here, each glycan was defined as a node, and two nodes were connected by an edge if there was at least 5% overlap between them (Fig. 8*B*). The only glycans from the neighboring protomers that were considered are those with an interprotomer edge. Fig. 8*C* shows the network obtained for the Man9 ensemble unfolded in two dimensions. It can be seen that overall there are three main regions of overlap—the V1/V2 apex, the gp41 base, and the densely occupied gp120 outer and inner domains that includes the high-mannose patch. The interprotomer overlaps are contributed mainly by the V1/V2 glycans. The nomenclature was inspired by the established nomenclature for gp120 domains (34); however, our definitions were adapted to better capture glycan shield structure (*SI Appendix*, Fig. S10*A*). The analysis was repeated for the Man5 ensemble and we observed a reduced overall connectivity as measured by the mean node degree (from ∼7 to ∼5) and the maximum network diameter (from five to eight hops), consistent with their smaller size (*SI Appendix*, Fig. S11).

With a network in place, we could then analyze the relative influence of each glycan on the whole system and examine its long-range structure. To do this, we calculated the relative eigenvector centrality of the nodes, which is a measure of importance in the network, and the results are projected on the network as a colormap in Fig. 8*C*. We see that the gp120 outer domain glycans at N332, N339, N363, N386, and N392, which are densely connected within the network, have strong eigencentrality measures. In the apex region, the V1/V2 loop glycans at N133, N160, and N185e/h also have relatively high eigencentrality due to their increased flexibility, while the glycans at N88, N234, N276, and those in gp41 have low eigencentrality, reflecting low interaction probabilities. Incorporated intrinsically into the network is a set of stable subgraphs that represent highly connected glycan clusters. To illustrate this structural hierarchy, we progressively stripped the network using tighter overlap cutoffs (*SI Appendix*, Fig. S10*B*). As the network is degraded, we see the formation of two large subgraphs, one composed of the V1/V2 apex and the gp120 outer domains and the second composed of the gp120 inner domain along with gp41 base glycans. With an even stricter cutoff, the sparsely connected glycans with low eigencentrality separate out and the two subgraphs split again to form four subgraphs: a V1/V2 apex domain, a gp120 outer domain, a gp120 inner domain, and, lastly, the group of unconnected gp41 base glycans.

### Highly Connected Glycan Clusters Are Resistant to Enzymatic Digestion and Help Stabilize the Prefusion Trimer.

**Fig. 9.**
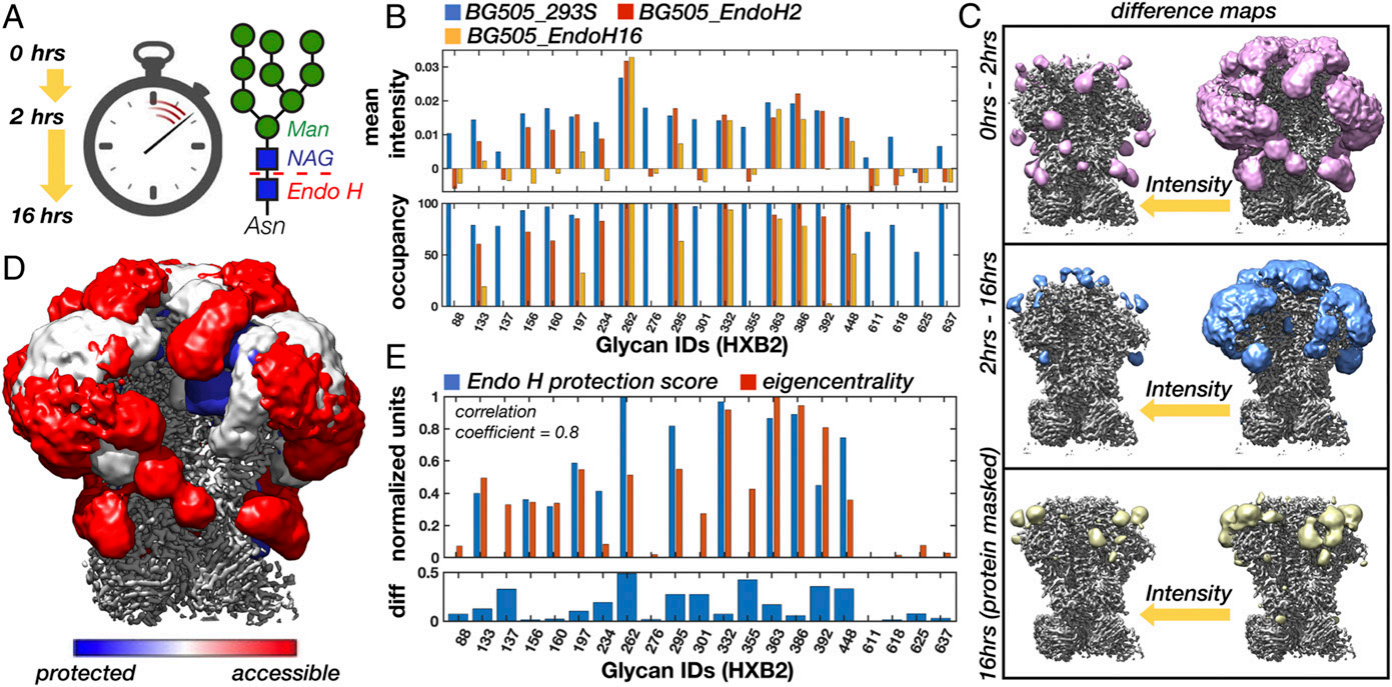
Time-resolved cryo-EM reveals that highly connected glycan clusters are resistant to enzymatic digestion. (*A*) Cartoon schematic of the Endo H digestion of high-mannose glycans and an illustration of the three reaction times captured by time-resolved cryo-EM. (*B*) Mean intensity around glycan BMA residues for the BG505_293S, BG505_EndoH2, and BG505_EndoH16 cryo-EM maps along with the percent occupancy at each site. Occupancy was calculated assuming a linear relationship between occupancy and intensity with the initial occupancy determined by MS. (*C*) BG505_293S − BG505_EndoH2 and BG505_EndoH2 − BG505_EndoH16 difference maps at two intensity thresholds. Difference maps were first multiplied by a soft mask around the RM20A3 Fabs and smoothed with a 2-SD Gaussian filter. Also shown is the residual glycan signal remaining after 16 h of digestion isolated by masking out the protein density. (*D*) Overlay of the three maps from *B* colored according to susceptibility to Endo H digestion. (*E*) Normalized Endo H protection score measured as the cumulative occupancy at each site in the two digestion intermediates and the normalized eigencentrality from the Man9 volume overlap network. Pearson correlation coefficient of ∼0.8 (*P* = 1.14e-05).

To determine if the glycan shield affects Env structure and dynamics, we exposed BG505_293S (already in complex with the RM20A3 Fab) to digestion by endoglycosidase H (Endo H) (Fig. 9*A*), and collected cryo-EM data on the deglycosylated sample. We hypothesized that highly connected glycans would be protected from enzymatic digestion, and vice versa. Therefore, if we could measure the order in which glycans were cleaved by Endo H it could provide indirect validation of our network models. To test this, we collected cryo-EM data on a partially deglycosylated sample as well. The reaction times and Endo H concentrations for achieving partial (2 h) and complete (16 h) digestion were determined prior to performing cryo-EM. The datasets, referred to as BG505_EndoH2 and BG505_EndoH16, reconstructed to ∼3.2- and ∼3.5-Å resolution, respectively, with similar overall quality, resulted in nearly identical atomic models to the other three datasets (*SI Appendix*, Fig. S1 *A* and *B*). The progressive digestion of the glycan shield was confirmed by sodium dodecyl sulfate-polyacrylamide gel electrophoresis and size-exclusion chromatography (*SI Appendix*, Fig. S12), while scale-space analysis of the cryo-EM maps revealed a clear progressive reduction of glycan signal (*SI Appendix*, Fig. S3 *G*–*I*).

Indeed, analysis of local map intensity around each glycan revealed that digestion occurred nonuniformly (Fig. 9 *B* and *C*). This was also confirmed by changes in the 3-D variability maps (*SI Appendix*, Fig. S9 *D* and *E*). By assuming a linear relationship between intensity and occupancy and using the MS data to set the initial occupancy, we calculated the occupancy at each site (Fig. 9*C*). After 2 h the gp41 glycans (N611 to N637) were completely digested, while some glycans, particularly those in the densely packed gp120 outer domain, remained mostly intact. Although we could not identify unique signal for the individual V2, V4, and V5 loop glycans, there was clear signal around these sites in the 0- to 2-h difference map only (Fig. 9*B*), indicating the dynamic V loop glycans were completely digested within 2 h. We also found partial signal reduction at a few sites, indicative of nonuniform digestion, for example the apex cluster composed of the N156 and N160 glycans as well as the glycans at N133, N197, and N234. After 16 h the glycan shield was almost completely digested; however, we still detected some residual glycan signal at the previously discussed cluster composed of the N363, N386, and N197 glycans, as well as a cluster composed of the N295, N332, and N448 glycans. In addition, the highly protected glycan at N262 remained completely intact. By quantifying the degree of protection from Endo H (*Materials and Methods*) and comparing it with the predicted network eigencentralities for the Man9 ensemble (Fig. 9*E*), we obtained a correlation coefficient of ∼0.8 (*P* = 1.14e-05), suggesting highly connected glycans are resistant to enzymatic digestion. Also evident is the similarity between the persistent glycan clusters and the subgraphs generated by progressively stripping the glycan overlap network (*SI Appendix*, Fig. S10*B*), which could be seen as mimicking the gradual digestion by Endo H.

**Fig. 10.**
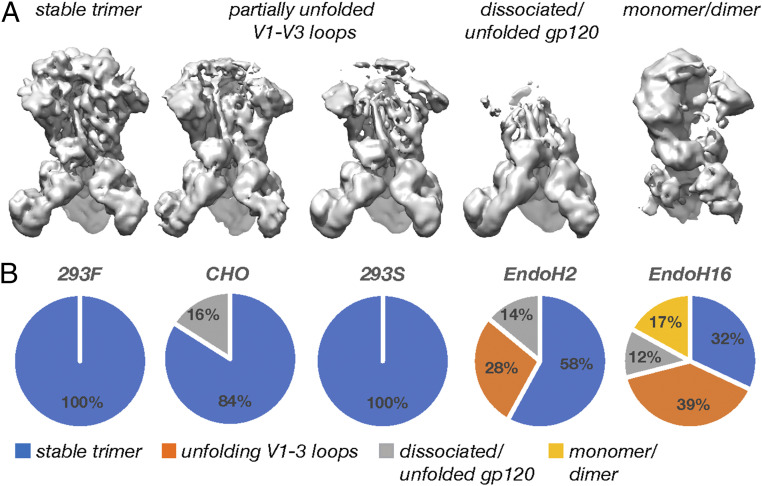
Digestion of the glycan shield leads to progressive destabilization of the Env trimer. (*A*) Representative cryo-EM maps of the unfolding intermediates captured by 3-D classification. (*B*) Pie charts showing the relative percentages of each class present in the five cryo-EM datasets presented here.

Interestingly, 3-D classification of the Endo H-treated datasets revealed an increasing degree of protein unfolding and subunit dissociation initiating at the V1 to V3 loops in the trimer apex. Classification consistently converged to four classes that appeared to capture heterogeneous intermediates in the unfolding process (Fig. 10*A* and *SI Appendix*, Figs. S13–S17). We interpreted these as representing 1) stable trimers, 2) trimers with unfolded or partially unfolded V1 to V3 loops, 3) trimers with fully unfolded/dissociated gp120s, and 4) monomers/dimers. As the reaction progressed, the percentages of unfolded trimers increased, while the percentage of stably folded trimers decreased (Fig. 10*B*). Although it cannot be easily confirmed, the unfolded trimers in each dataset were likely more completely deglycosylated than the particles that make up the stable trimeric classes. We also observed a small percentage of dissociated gp120s in the CHO sample, perhaps as a result of the higher degree of suboccupancy. When viewed in context of the results presented above, we concluded that the highly connected glycans and glycan clusters that were resistant to digestion are also critical to maintaining structural stability of the prefusion Env trimer, suggesting the glycan shield may have additional functions beyond its role in immune evasion.

## Discussion

Prior to this study, there were conflicting reports of glycan shield structure from cryo-EM and X-ray crystallography measurements (6, 14, 35). Here, we did not observe any stabilized glycan–glycan interactions like those reported in crystal structures (6), suggesting methodological differences may be responsible for the apparently contradictory results. For example, the crystal structures included two bound broadly neutralizing antibodies (bnAbs) per monomer to facilitate crystal packing, both of which engage multiple glycans and disrupt the native dynamics and higher-order structure. Desolvation of mobile waters embedded within the glycan shield during crystallization could also potentially induce the stabilized glycan–glycan contacts observed. A favorable interpretation that also fits with the conclusions of this paper is that the crystal structures captured artificially stabilized yet physiologically relevant conformations and are thus suggestive of the types of transient noncovalent interactions possible between glycans.

Our work represents a case of using cryo-EM to validate glycoprotein ensembles and, in turn, their use in guiding cryo-EM analysis. We found that the HT-AM pipeline captured physiologically relevant glycan sampling at global and local scales, however deviated from the experimental observations when the flexibility of the underlying protein was greater than could be captured by the current pipeline. Thus, exploring methods to enhance sampling of the protein backbone, particularly at Env variable loops, represents a clear route for improving the accuracy of the pipeline (36). Another logical next step is to use the experimental cryo-EM maps to steer the ensemble modeling process, which has been successful for nonglycosylated proteins (37–39); however, extending such techniques to capture the extreme levels of heterogeneity present in the glycan shield will be challenging. Experimentally, our ability to capture differences in glycan dynamics from cryo-EM maps is limited when using local map intensity around BMA residues alone, and such limitations could also be contributing to the observed discrepancies. Again, using the cryo-EM maps to steer the modeling process could provide more accurate and comprehensive assessments of variations in relative dynamics between glycans in addition to improving modeling accuracy. Finally, utilizing sequence engineering and/or different expression systems to achieve a more uniformly glycosylated Env sample or, conversely, incorporating experimentally relevant levels of heterogeneity into the modeling pipeline will allow for a more accurate comparison between theory and experiment.

Using graph theory, we mapped out the complex network of potential interactions within the glycan shield. A similar network-based approach was introduced previously to capture concerted behavior within the glycan shield during all-atom MD simulations (22). Although the HT-AM pipeline does not capture temporal dynamics, we found that the probabilistic networks reported here closely resembled the ones derived from the MD simulations, and the two lead to similar predictions, for instance, the clustering of glycans into larger microdomains (*SI Appendix*, Fig. S10). Moreover, the overlap probability between glycans gives a measure of the structural shielding rather than the energetic interactions between glycans. At a qualitative level, the hierarchy of structural features we observed in the Gaussian filtered and 3-D variability maps (Fig. 2 *C* and *D*) could be seen as the ensemble-averaged manifestation of this clustering behavior. Given the preference of bnAbs for targeting the interfaces between higher-order glycan clusters (22), experimentally mapping such long-range structure could be important. At a more quantitative level, we found that the average degree of protection from Endo H digestion correlated strongly with network eigencentrality for those glycans we could identify in the cryo-EM maps, and that the order of digestion closely matched the order in which glycans were removed from the network when applying stricter overlap thresholds. It should be noted, however, that because of the somewhat simplified approach we have taken to modeling the most dynamic segments of the variable loops, in particular in the V4 loop segment containing the N398, N406, and N411 glycans, the eigencentrality of these glycans may be artificially high, leading to minor discrepancies between digestion susceptibility and network centrality. Nonetheless, these correlations could be seen as providing indirect validation of the network models, where the high-centrality glycans are either located in densely packed regions Endo H would have a difficult time accessing (*SI Appendix*, Fig. S6 *D* and *E*) or are protected by strong interaction probability with their neighbors.

We demonstrated that cryo-EM is capable of detecting and quantifying changes in glycan dynamics, as well as occupancy and chemical composition between differentially glycosylated Envs, the latter of which were exclusively provided by MS prior to this study. Thus, cryo-EM provides validation for these measurements while contributing insight into the structural impact of changes in glycosylation. Importantly, we found evidence for more extensive changes in glycosylation between the 293F- and CHO-expressed samples than detected by MS, which has clear relevance to the currently ongoing human clinical trials based on the CHO-expressed Env. In addition to these results, we found that the extent of processing measured by the percentage of high-mannose–type glycans at each site was strongly correlated with ordering in the cryo-EM maps (*SI Appendix*, Fig. S2 *C* and *D*). The relationship between these variables likely reflects their mutual dependence upon glycan crowding (*SI Appendix*, Fig. S6 *B* and *C*), as well as the properties surrounding protein structure, both of which can reduce dynamics and restrict the access of Endo H and glycan-processing enzymes in the cell.

The observation that enzymatic deglycosylation leads to progressive destabilization of the Env trimer was somewhat surprising because recent investigations into the effect of glycan knockouts and deglycosylation on Env stability and infectivity have come to different conclusions (40–44). However, there is ample biophysical evidence that glycans influence protein stability, dynamics, and folding, even in the case of Env (17, 45–56), although it is not obvious which mechanisms are important here. It has been proposed that glycans stabilize proteins primarily by destabilizing the unfolded state and by increasing the height of the unfolding energy barrier (47). The same study also found that glycosylation reduced the entropy of the folded state, similar to the effects of molecular crowding or confinement, although the effect was canceled out by an equivalent change in enthalpy. Along these lines, the slightly lower resolution of the BG505_EndoH16 map compared with the fully glycosylated maps could be indicative of increased dynamics. In addition, our observation that the Man9 glycans were less dynamic than the smaller Man5 glycans in our simulations could be attributed to more crowding in the canopy, which in turn could further stabilize the underlying protein. Also, the stabilizing effects were found to depend strongly on the number of glycans as well as their positions on the protein (47), which is in line with the results of our Endo H digestion experiment. Therefore, given the high density of glycans on the surface of Env and the importance of their tertiary arrangement into high-density clusters, it is plausible these stabilizing effects could be amplified. Furthermore, glycan–glycan interactions such as those observed in the crystal structures solved by Stewart-Jones et al. (6) could also play a role in stabilizing the folded state. Although our results clearly show that such interactions must be more dynamic than the crystal structures suggest, the combined effect of many such short-lived interactions could become significant.

The time-resolved Endo H digestion experiment provides an explicit mapping of the importance of each glycan toward maintaining the integrity of the glycan shield and the overall stability of Env. In addition to the glycan at N262, the two highly connected clusters in the gp120 outer domain centered around the N386 and N295 glycans were the most important for trimer stability, and to a lesser extent the apex cluster composed primarily of the N156 and N160 glycans. In support of this, previous studies have shown that knocking out the glycans at N386 or N262 individually has dramatic effects on trimer stability (33), whereas deletion of up to five glycans at once peripheral to the CD4 binding site (CD4bs), which are not part of these clusters, is well-tolerated (10, 57, 58). In addition, the glycans at N386 and N295 in particular were found to strongly control the processing of the other glycans near them, reinforcing their importance in maintaining the integrity of large-scale glycan shield structure (33). These results have implications for rational vaccine design where removing certain glycans could have undesired effects on protein stability and suggest that there are limits to the number of glycan deletions that can be tolerated, with particular emphasis on maintaining the integrity of the aforementioned clusters. In addition, we found that instability originated at the trimer apex at the V1 to V3 loops. This region is known to be metastable and to change conformation upon CD4 binding (34, 59). Therefore, it is plausible the glycans and glycan clusters in this region are contributing to maintaining Env in a metastable state poised for receptor binding. Given the high conservation of the glycan clusters identified here, our results are likely universal to all Env sequences. Lastly, it is possible that glycan-induced stabilization of Env may enable hypermutation of the underlying protein surface, thereby endowing Env with the ability to escape immune pressure with otherwise deleterious mutations.

**Fig. 11.**
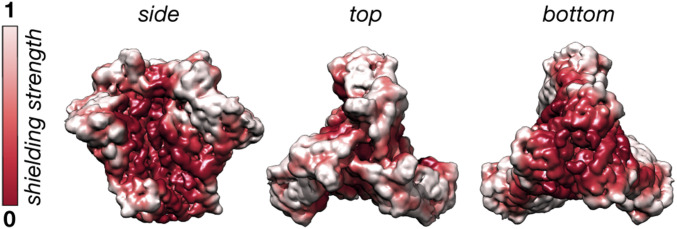
Quantifying the glycan shielding effect from cryo-EM maps. The normalized glycan shielding effect for BG505_CHO defined as the total glycan signal within an ∼7-Å-radius spherical probe centered at each voxel. White surfaces are strongly shielded by glycans.

It is known that immune responses to Env preferentially target glycan-depleted surface area (9, 10), and the results presented here provide experimentally determined mapping of this surface. The dynamics of the glycans create a cloud/shield over the protein, and thus an approaching antibody will effectively “see” this blurry ensemble-averaged structure, which is consistent with the relatively slow on rates of most HIV bnAbs. We quantified the shielding effect with a simple “rolling sphere” method for BG505_CHO and visualized the results as a colormap projected onto the surface of Env (Fig. 11). White surfaces are strongly masked by nearby glycans and red surfaces are weakly masked, highlighting sites of potential vulnerability to neutralizing antibodies (see *SI Appendix* for details). Notably, the surface exposure revealed by these cryo-EM ensemble averages is consistent with the net serum antibody responses observed in immunization studies (60, 61).

Looking beyond the results of this paper, our integrated approach can be easily extended to other Env clones and to heavily glycosylated spike proteins from other viruses such as influenza, Ebola, Lassa, and coronaviruses, and represents a potentially powerful approach for studying the structure and dynamics of glycoproteins in general.

## Materials and Methods

BG505 SOSIP.664v3 was expressed and purified from HEK293F and HEK293S suspension cell culture in house while the CHO cell line-derived BG505 SOSIP.664 was provided to us by the IAVI and expressed and purified as described in Dey et al. (31). The monoclonal antibody RM20A3 was isolated from a BG505 SOSIP.664-immunized rhesus macaque (62) and the Fab was expressed and purified from HEK293F cells as described previously (63). The BG505 SOSIP.664 samples expressed in HEK293F and CHO cell lines were prepared for MS analysis as described previously (25), and the HEK293S sample was prepared with slight modifications on that protocol. All samples were prepared for cryo-EM analysis via the same protocol after being complexed with the RM20A3 Fab. Imaging was performed on either an FEI Titan Krios or Talos Arctica (Thermo Fisher) microscope. All noncustom cryo-EM data-processing steps were performed using a combination of RELION-2/3 (64, 65) and cryoSPARC v1 (66). Model building and refinement were carried out with UCSF Chimera (67), Coot (68, 69), and Rosetta (70). All custom analysis of cryo-EM maps was performed in MATLAB (2018b) (MATLAB and Image Processing Toolbox Release 2018b, The MathWorks). Three-dimensional variability analysis was performed in SPARX (27). The HT-AM pipeline was carried out by implementing the ALLOSMOD (29, 71) package of MODELLER (72, 73) in a streamlined pipeline. All graph theory and network-based analysis was performed using Python (Python Software Foundation; https://www.python.org/) and MATLAB_R2018a packages. Simulated cryo-EM data generation and analysis were performed in RELION. All cryo-EM map and model visualization was performed with UCSF Chimera. A comprehensive description of all of the methods can be found in *SI Appendix*.

## Supplementary Material

Supplementary File

## Data Availability

All data needed to evaluate the conclusions in this paper are present in the paper and/or *SI Appendix*. Atomic models and electron potential maps with their corresponding half maps, masks, and SPARX 3-D variability maps, have been deposited to the Protein Data Bank and Electron Microscopy Data Bank respectively under the following accession codes: BG505_293F (6X9R; EMD-22108), BG505_CHO (6X9S; EMD-22109), BG505_293S (6X9T; EMD-22110), BG505_EndoH2 (6X9U; EMD-22111), BG505_EndoH16 (6X9V; EMD-22112).
